# Incidence and predictors of radial artery occlusion after transradial coronary catheterization

**DOI:** 10.1186/s43044-019-0008-0

**Published:** 2019-09-05

**Authors:** Mohamed A. Sadaka, Waleed Etman, Walid Ahmed, Saeed Kandil, Salah Eltahan

**Affiliations:** 1 Cardiology and Angiology Department, Faculty of Medicine, Alexandria University, Alexandria, Egypt; 2 Cardiology and Angiology Department, Medical Research Institute, Alexandria University, Alexandria, Egypt

## Abstract

**Background:**

Radial artery occlusion (RAO) is considered the most common and devastating complication of transradial approach (TRA). It has been described as the “Achilles’ heel” of the transradial technique. Our aim was to assess the incidence and predictors of radial artery occlusion after transradial coronary catheterization.

**Results:**

This was a prospective study enrolling 164 patients undergoing percutaneous coronary interventions (PCI) via the transradial approach (TRA) using 6-F catheters. Doppler ultrasonography assessment of the radial artery (RA) was conducted on day 1 and 6 months following the procedure. The studied group included 104 male (63.4%) and 60 female (36.6%) patients with a mean age of 57.7 ± 8.8 years and a mean RA diameter of 2.8 ± 0.5 mm. On day 1, Doppler examination revealed RAO in 54 patients (32.9%). After 6 months, RAO was detected in 49 patients (29.9%). Interestingly, only 1 new case (0.9%) of RAO was noted, and 6 patients (11.1%) had regained their RA patency. On multivariate analysis, female gender, age, manual compression, and RA diameter emerged as independent predictors of RAO. Using TR band for hemostasis for only 2 h was recognized as a potent independent predictor of RA patency on day 1 and 6 months after the procedure (*n* = 2, 3.7% in the RAO group, vs. *n* = 23, 20.9% in the non-RAO group, *p* = 0.004).

**Conclusion:**

RAO, although clinically a silent issue, has been the main complication following TRA. In patients with high predictors of RAO, careful management and close follow-up are required to ensure radial artery long-term patency.

## Background

Coronary artery disease (CAD) has been considered one of the major causes of morbidity and mortality in developed countries. Although mortality rates due to CAD worldwide have declined over the past seven decades, yet it is still responsible for about one third or more of all deaths in individuals over the age of 55 years [1].

Coronary angiography has become the gold standard for the diagnosis and establishing treatment strategies for atherosclerotic coronary artery disease [2].

Coronary angiography can be performed via the femoral, radial, or ulnar arteries. The common femoral artery has long been the access site of favor for doing coronary angiography and angioplasty. However, vascular access site complications such as bleeding, hematoma, arteriovenous fistula, or pseudoaneurysm are not uncommon after transfemoral approach (TFA) procedures [3].

Bleeding complications are associated with an increased risk for death, myocardial infarction (MI), stroke, stent thrombosis, and increased expenses [4].

The majority of bleeding complications in patients undergoing percutaneous coronary intervention (PCI) are related to the vascular access site [5]. The reduction of ischemic events and complications after PCI increases the importance of vascular access site and bleeding complications, which were shown to be correlated with morbidity and mortality [6].

The transradial approach (TRA) was first introduced by Campeau in 1989 for diagnostic coronary angiography [7]. Afterwards, it was subsequently improved upon by Kiemeneij and Laarman for PCI [8].

The radial artery is anatomically more accessible than the femoral artery and, therefore, more easily compressible [9]. TRA offers advantages in comparison with transfemoral (TF) access including decreased incidence of access site bleeding, especially under conditions of aggressive anticoagulation and antiplatelet treatment, earlier ambulation, and improved patient comfort [10]. And due to the previously mentioned privileges, the European Society of Cardiology Guidelines on Myocardial Revascularization recommended the use of radial access as the standard approach for coronary angiography and angioplasty, provided that no overriding procedures are required [11].

Although operators are gaining more and more experience in the TRA, this has led to decreased experience in TFA, leading to more access site complications when the latter is chosen, the so called Campeau Radial Paradox [12].

Radial artery occlusion (RAO) is considered the most common and devastating complication of TRA. It has been described as the “Achilles’ heel” of the TR technique [13]. The reported incidence of such complication ranges between 0.8 and 30% [14].

Despite the fact that RAO may carry an asymptomatic course due to the presence of extensive collateralization of the hand through the palmar arch and forearm arterioles which prevent hand ischemia, it eliminates the ability to reuse the radial artery for future purposes. A patent radial artery can be recannulated for future coronary artery procedures; intra-arterial pressure monitoring, used as a conduit for coronary artery bypass grafting; or arteriovenous fistula formation for hemodialysis in patients with end-stage renal disease [15, 16].

The aim of the study is to assess the incidence of RAO immediately after TR procedure and in long-term follow-up and to evaluate its predictors.

## Results

### Study population

This is a prospective study which included 164 patients who underwent TR elective diagnostic coronary study and elective PCI from January to May 2018. Patients with severe sepsis, local site infection, and previous contrast allergy were excluded from the study.

### Coronary angiography and angioplasty

The wrist was sterilized and draped in the usual fashion. After local subcutaneous anesthesia with 2–3 ml of 2% lignocaine, the RA was punctured with a 21-G needle and a 6-F sheath was introduced into the artery, using the Seldinger technique. After sheath insertion, 5000 IU unfractionated heparin (UFH) was given through the side arm of the sheath. For patients undergoing PCI, UFH dosage was calculated according to the patient’s weight. In addition, vasodilator cocktail, nitroglycerine, verapamil, or both, was given through the side arm of the sheath. The sheath was removed immediately after the procedure, and hemostasis was achieved.

### Doppler ultrasonography of the RA

Radial artery Doppler ultrasonography was conducted immediately and at least 6 months following the procedure.

**Fig. 1:**
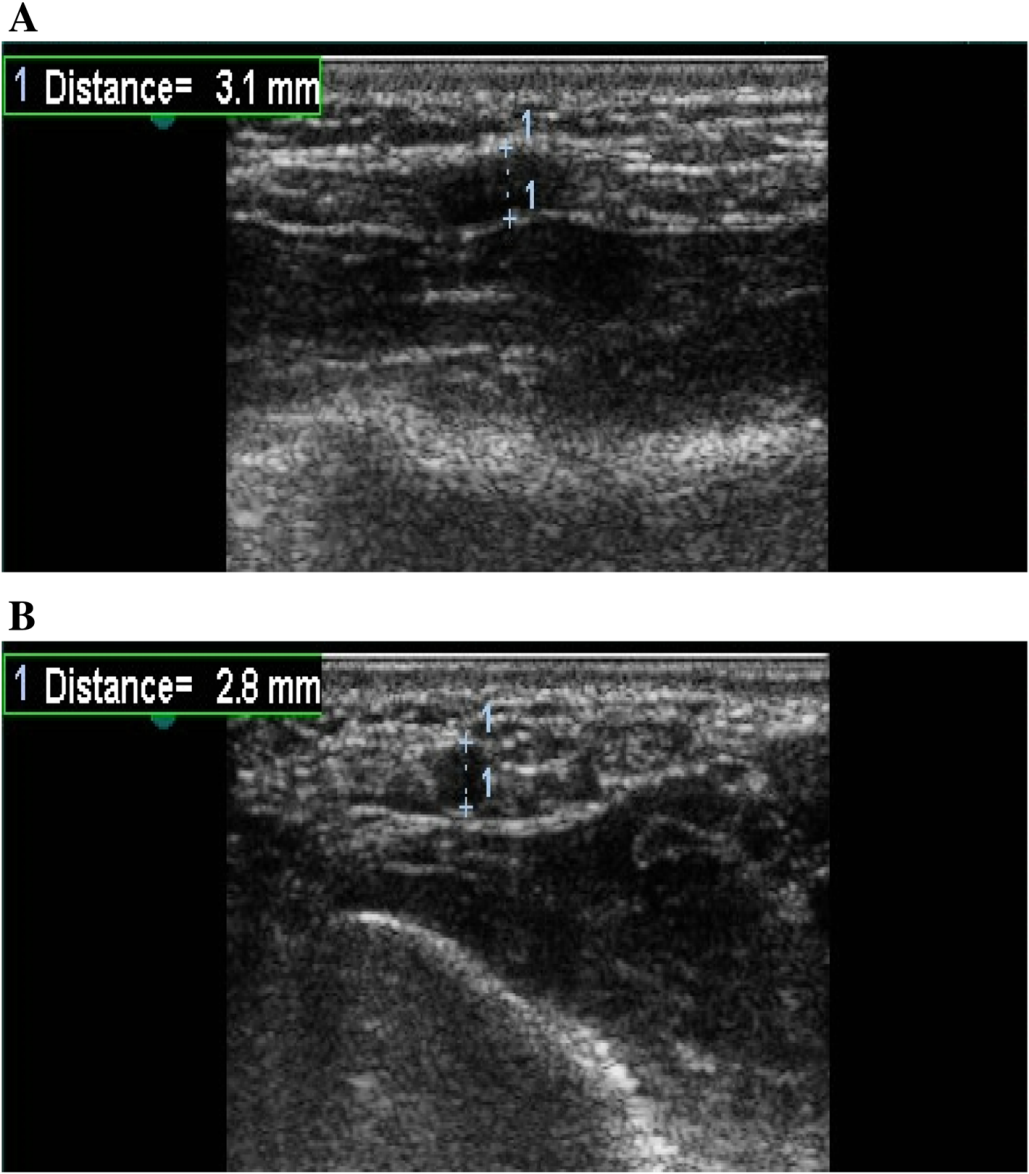
Ultrasonography of the RA using the B-mode. **a** Diameter of the RA in the horizontal section. **b** Diameter of the RA in the longitudinal section

**Fig. 2:**
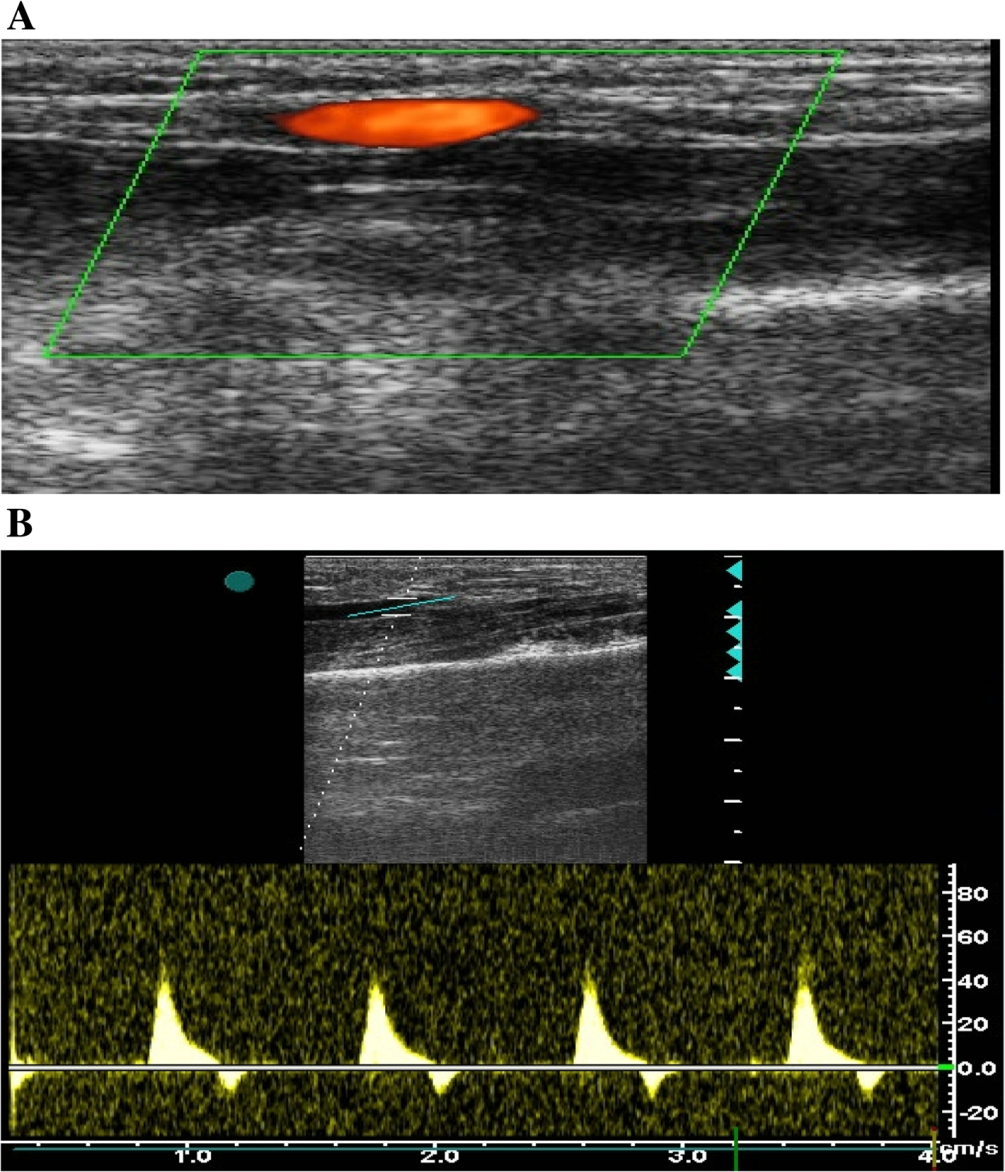
Duplex ultrasonography of a patent RA after TR coronary catheterization. **a** Color Doppler showing normal flow within the RA. **b** Spectral analysis showing tri-phasic flow pattern

**Fig. 3:**
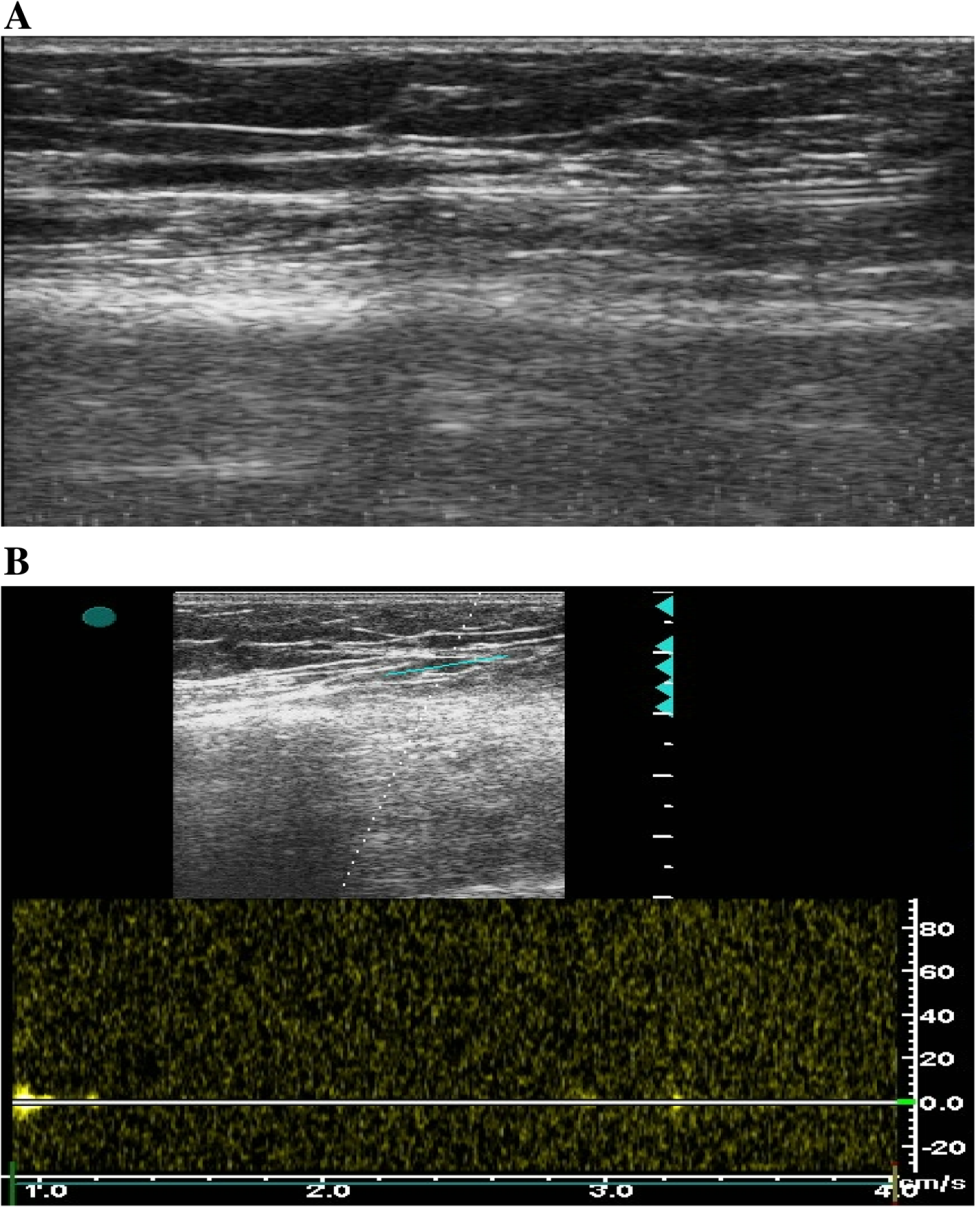
Duplex ultrasonography of an occluded RA after TR coronary catheterization. **a** B-mode showing total occlusion of the RA with thrombosis distal to the site of occlusion. **b** Spectral analysis showing no flow distal to the site of occlusion

All examinations were performed using either GE Vivid 3 or Philips Envisor by a 9–14-MHz multi-frequency matrix linear vascular probe. The ultrasound probe was placed on the ventral aspect of the ipsilateral forearm, 2 cm proximal to the radial styloid process. The color Doppler mode was used to localize the RA and assess its patency, in addition to flow velocity measurement by spectral analysis. The internal diameter of the RA (intima to intima) was to be measured using the two-dimensional sonography (B-mode). Figures 1, 2 and 3 show the different case examples in our study.

### Data collection

Demographic and patients’ clinical characteristics were collected. Medications used during the procedure, procedural time, local site compression method after sheath removal (i.e., method of hemostasis), any difficulties or complications faced during the procedure, and hand function at the time of discharge and 6 months later were recorded.

### Definitions

RAO was defined as the absence of radial pulsations on palpation and antegrade flow signal on Doppler studies.

Procedural time was defined as the time between local anesthesia and the removal of the last catheter.

Post-procedural pain was defined as the puncture site or forearm pain following hemostasis with or without swelling during the hospital stay or at out-hospital follow-up.

Major hematoma was defined as hematoma more than 5 cm in diameter, while minor one was defined as less than 5 cm in diameter.

Procedure success was defined as coronary angiography or angioplasty completed via the transradial route without changing to another vascular access.

### Statistics

Statistical analyses were performed using IBM SPSS software package version 20.0 (Armonk, NY; IBM Corp.) Qualitative data were described using number and percent. Quantitative data were described using range (minimum and maximum), mean, standard deviation, and median. Multiple logistic linear regression analysis was performed to identify the independent predictors of RAO. Continuous variables were compared using Student’s *t* test and Mann-Whitney *U* test, as appropriate. Differences between the categorical variables were examined using the chi-square test. Significance of the obtained results was judged at the 5% level.

### Demographic and clinical characteristics of the studied population

The studied population included 164 patients with a mean age of 57.7 ± 8.8 years. RA Doppler ultrasonography was conducted immediately and at least 6 months after the procedure with a mean follow-up period of 6.3 ± 1.4 months. On day 1, Doppler examination revealed RAO in 54 patients (32.9%). After 6 months, RAO was detected in 49 patients (29.9%); only 1 new case (0.9%) of RAO was noted, and 6 patients (11.1%) had regained their RA patency. The mean RA diameter in the studied group was 2.8 ± 0.5 mm. Table 1 summarizes the demographic and clinical characteristics of the studied population.

**Table 1:** Demographic and clinical characteristics of the studied patients (*n*=164)

Age (years)	57.7 ± 8.8
Gender, n (%)	
Male	104 (63.4)
Female	60 (36.6)
Body weight (kg)	86.3 ± 11.8
Height (cm)	171.6 ± 8.0
Body mass index (kg/m^2^)	29.2 ± 4.9
Risk factors, n (%)	
Smoking	71 (43.3)
Diabetes	84 (51.2)
Hypertension	87 (53.0)
Dyslipidemia	96 (58.9)
Family history of premature coronary artery disease	4 (2.4)
History of other cardiovascular diseases (example: stroke, peripheral vascular disease)	6 (3.7)
Previous RA cannulation, n (%)	4 (2.4)
RA diameter (mm)	2.8 ± 0.5
Radial artery occlusion, n (%)	54 (32.9)

### Demographic and clinical characteristics of the studied groups

According to day 1 RA Doppler ultrasonography, patients were divided into 2 groups: patients with occluded RA (RAO group) and patients with patent RA (non-RAO group). On day 1, Doppler examination revealed RAO in 54 patients (32.9%). After 6 months, RAO was detected in 49 patients (29.9%); only 1 new case (0.9%) of RAO was noted, and 6 patients (11.1%) had regained their RA patency. Table 2 summarizes the demographic and clinical characteristics of both groups. The RA diameter was positively correlated with the patient’s age (*r* = 0.337, *p* = <0.001) and height (r = 0.168, p = 0.031). Female patients were associated with statistically significant smaller RA diameters than those of male patients (2.6 ± 0.4 vs. 2.9 ± 0.4 mm, *p* ≤ 0.001). Smokers were associated with larger RA diameters than those of non-smoker which was statistically significant (2.9 ± 0.5 vs. 2.71 ± 0.41 mm, *p* = 0.006).

**Table 2:** Demographic and clinical characteristics of the study groups

	Non-RAO group	RAO group	P
	(*n*=110)	(*n*=54)	
Age (years)	59.4 ± 7.8	54.4 ± 9.7	0.001^*^
Gender, n (%)			
Male	83 (75.5)	21 (38.9)	<0.001^*^
Female	27 (24.5)	33 (61.1)	
Body weight (kg)	87.4 ± 12.4	84.2 ± 10.4	0.099
Height (cm)	172.4 ± 8.1	169.9 ± 7.7	0.069
Body mass index (kg/m^2^)	29.2 ± 4.6	29.2 ± 5.5	0.970
Risk factors, *n (%)*			
*Smoking*	48 (43.6)	23 (42.6)	0.899
*Diabetes*	55 (50.0)	29 (53.7)	0.656
*Hypertension*	63 (57.3)	24 (44.4)	0.122
*Dyslipidemia*	66 (60.6)	30 (55.6)	0.545
*Family history of premature coronary artery disease*	3 (2.7)	1 (1.9)	1.00
*History of other cardiovascular diseases (example: stroke, peripheral vascular disease)*	3 (2.7)	3 (5.6)	0.396
Previous RA cannulation, *n (%)*	2 (1.8)	2 (3.7)	0.599
RA diameter (mm)	2.9 ± 0.4	2.5 ± 0.5	<0.001^*^

*RA* Radial artery, *RAO* Radial artery occlusion

^*^Statistically significant

### Procedural data and method of hemostasis of the studied groups

Table 3 summarizes the procedural data for non-RAO and RAO groups. TR coronary catheterization was successfully completed in all patients. There were no major bleeding episodes, pseudo-aneurysm, or arteriovenous fistula formation. Despite that arterial spasm developed in nine cases (5.5%), this was a transient event and did not affect the course of the procedure. Hematoma developed in seven cases (4.3%) which were all minor. Various methods of puncture site compression were used. All patients were asymptomatic except for three patients (1.8%) who complained of post-procedural pain which was managed by hot compresses and small doses of NSAIDs. Hence, the RAO group was asymptomatic in almost all patients and has no impact on hand functions because of good ulnar artery flow; no specific management was utilized except subcutaneous enoxaparin 60 mg twice daily for 3 days. Table 4 summarizes the method of hemostasis used for both studied groups.

**Table 3:** Procedural data of the study groups

	Non-RAO group (*n*=110)	RAO group (*n*=54)	P
Procedure time (min)	24.43 ± 26.36	22.85 ± 16.40	0.434
Puncture site, n (%)			
Proximal	93 (84.5)	48 (88.9)	
Distal (snuff-box approach)	17 (15.5)	6 (11.1)	0.452
UFH given, n (%)			
IV	45 (40.9)	15 (27.8)	
Inside the sheath	65 (59.1)	39 (72.2)	0.101
NTG given, n (%)	74 (67.3)	36 (66.7)	1.000
Verapamil given, n (%)	104 (94.5)	51 (94.4)	1.000
Number of catheters used	1.7 ± 0.9	2.2 ± 1.4	0.032^*^
Multiple radial punctures, n (%)	9 (8.2)	3 (5.6)	0.752
Resistance during sheath insertion, n (%)	1 (0.9)	2 (3.7)	0.252
Arterial spasm, n (%)	5 (4.5)	4 (7.4)	0.478
Hemodynamically compromising events (such as ventricular arrhythmia or sudden drop in blood pressure), n (%)	1 (0.9)	5 (9.3)	0.015^*^
Minor hematoma, n (%)	6 (5.5)	1 (1.9)	0.427
Post-procedural pain, n (%)	2 (2.0)	1 (2.0)	1.000

*NTG* Nitroglycerine, *RA* Radial artery, *RAO* Radial artery occlusion, *UFH* Unfractionated heparin

^*^Statistically significant

**Table 4:** Method of hemostasis used for the study groups

	Non-RAO group (*n*=110)	RAO group (*n*=54)	P
Manual RA compression, n (%)	32 (29.1)	34 (63.0)	<0.001^*^
TR band for 2 hours, n (%)	23 (20.9)	2 (3.7)	0.004^*^
TR band for 6 hours, n (%)	17 (15.5)	5 (9.3)	0.274
Direct rolled gauze compression, n (%)	38 (34.5)	13 (24.1)	0.173

*RA* Radial artery, *RAO* Radial artery occlusion

^*^Statistically significant

### Predictors of RAO

Female patients had a higher incidence of RAO than male patients (Table 2). In addition, RAO occurred more in patients with smaller RA diameter (2.5 ± 0.5 mm in the RAO group vs. 2.9 ± 0.4 mm in the non-RAO group, *p* < 0.001).

Moreover, the number of catheters used was more in the RAO group (2.2 ± 1.4 catheters in the RAO group vs. 1.7 ± 0.9 catheters in the non-RAO group, *p* = 0.032). Patients with RAO had more hemodynamically compromising events than those with patent RA (Table 3).

On multivariate analysis, female gender, age, manual compression ,and RA diameter emerged as strong independent predictors of RAO. On the other hand, using TR band for hemostasis for only 2 h was recognized as a potent independent negative predictor of RAO on day 1 and 6 months after the procedure (*p* = 0.004). The relative risk for RAO decreased to 0.124 (95% CI 0.013–1.217).

## Conclusion

Compared with the previously declared literature [15–18], this study showed a higher incidence of RAO (32.9%). This discrepancy can be attributed to several causes.

First of all, the diagnosis of RAO in our study was Doppler-based compared to previous studies in which RAO was diagnosed by the absence of RA pulsations [19–21]. Even in the presence of proximal RAO, pulsations may still be strongly felt due to the presence of collateral circulation from the palmar arch. By relying on an objective technique like duplex ultrasonography, false-positive results can be easily eliminated, and thus, underestimation of RAO can be avoided.

Secondly, different methods of hemostasis were used to observe its impact on RAO. Manual compression emerged as a significant predictor for RAO. Tight aggressive prolonged compression leads to a complete cessation of blood flow and eventually to thrombus formation. On the other hand, TR band compression for 2 h was a negative predictor for RAO. This drew our attention towards the significance of the hemostatic compression time. Only 2 h compression using TR band was recognized as a potent independent predictor of RA patency (*p* = 0.004) compared to TR band compression for 6 h (*p* = 0.274) which was associated with statistically non-significant difference.

Pancholy and Patel [22] discovered that shorter hemostatic compression durations were associated with a significantly lower incidence of early and chronic RAO without any increase in bleeding complications. The potential explanation for such a result is that the anticoagulant effect of UFH is expected to last for approximately 4 h. Compression periods longer than 4 h will subject the patient to a higher risk of thrombosis. However, after further and deeper investigations, they found that maintaining RA patency during hemostasis was the most significant protective predictor against RAO. Implying that if the blood flow of the RA is maintained during hemostasis, longer durations of compression can be tolerated without a higher risk of RAO. Thus, compression duration lost its significance provided that RA patency is maintained during compression.

In the RACOMAP study (radial compression guided by mean artery pressure versus standard compression with a pneumatic device), Cubero et al. [23] concluded that using pneumatic compression guided by mean artery pressure was associated with significantly lower risk of RAO than the conventional method, due to the accurate measurement of pressure exerted on the RA and thus avoiding total arterial collapse.

Ognerubov et al. [24] conducted a study to evaluate the ability of recanalization of acute occlusion of the RA after coronary angiography by applying a compression bandage to the ipsilateral ulnar artery. They discovered that not only the compression duration significantly affects the incidence of RAO; it also has a significant impact on its treatment. The success of ipsilateral ulnar artery compression to achieve recanalization of the RA was significantly lower in case of prolonged compression bandage use.

In our study, all the catheters and vascular sheaths used in the conducted patients were 6-F. For a given fixed catheter and sheath outer diameter (2 mm), RA diameter emerged as a significant independent short- and long-term predictor of RAO. Patients with a mean RA diameter of 2.5 ± 0.5 mm were at a higher risk of RAO. Smaller RA caliber was associated with a significant compromise in the blood flow after insertion of the sheath. Moreover, the stretching effect caused by the mismatch of a relatively larger sheath in relation to the RA diameter can provoke spasm leading to further vascular damage in the form of intimal flaps and triggering the cascade of intimal hyperplasia and remodeling with future occlusion of the vessel. Dahm et al. [25] conducted a randomized control trial to compare 5-F and 6-F guiding catheters in PCI. They revealed that 5.9% of patients in the 6-F group had RAO, while only 1.1% in the 5-F group had their RA occluded which was statistically significant (*p* = 0.05). Therefore, they affirmed on the fact that the sheath to artery ratio < 1 was a potent predictor of RA patency. This emphasizes that both RA diameter and sheath diameter should not be considered as crude numbers, but the relation between both diameters and the ratio between them should be always put in mind.

The number of catheters used in the RAO group was more than that used in the non-RAO group which represented a statistically significant difference (Table 3). Repeated introduction of the guidewire and angiographic catheters can provoke RA spasm. Although this can be minimally prevented by using a long guidewire (260 cm), the repeated introduction and withdrawal of catheters can easily damage the arterial intima leading to thrombosis or even dissection and perforation of the RA and subsequently its occlusion.

In the present study, there was no statistically significant difference concerning the complications faced during the procedure except for hemodynamically compromising events at the short-term follow-up only. During such events, the low blood pressure suffered by the patients, either vagally mediated or due to arrhythmia, leading to blood stasis together with an intimal injury of the RA provides an excellent milieu for the activation of the coagulation cascade and subsequently thrombosis. When resuscitation measures are taken, blood pressure begins to build up to its baseline values, recanalization of the RA and reestablishment of the blood flow are achieved. So, we can consider that hemodynamic instability is only a short-term predictor of RAO once the condition is stabilized and the obstacle of low blood pressure has been overcome.

Another risk factors for RAO were young and female patients which can be attributed to smaller RA diameters in such population. Patient’s age is associated with a statistically significant positive correlation with the RA diameter (*r* = 0.337, *p* ≤ 0.001). We speculate that the aging process is associated with ischemic pre-conditioning leading to larger RA diameters in the older population. Lee et al. [26] investigated the incidence and predictors of RAO after using sheathless standard guiding catheters in complex coronary intervention and carotid artery stenting by TRA and found that patients with RAO were younger than those with patent RA (56.0 ± 17.6 years in patients with RAO vs. 65.7 ± 10.4 years in patients without RAO, *p* = 0.032).

## Data Availability

All the data and materials utilized in this research are available.
